# Inter‐tissue and inter‐organ signaling in drought stress response and phenotyping of drought tolerance

**DOI:** 10.1111/tpj.15619

**Published:** 2021-12-16

**Authors:** Takashi Kuromori, Miki Fujita, Fuminori Takahashi, Kazuko Yamaguchi‐Shinozaki, Kazuo Shinozaki

**Affiliations:** ^1^ Gene Discovery Research Group RIKEN Center for Sustainable Resource Science 2‐1 Hirosawa Wako Saitama 351‐0198 Japan; ^2^ Gene Discovery Research Group RIKEN Center for Sustainable Resource Science 3‐1‐1 Koyadai Tsukuba Ibaraki 305‐0074 Japan; ^3^ Department of Biological Science and Technology Graduate School of Advanced Engineering Tokyo University of Science 6‐3‐1 Niijyuku, Katsushika‐ku Tokyo 125‐8585 Japan; ^4^ Laboratory of Plant Molecular Physiology Graduate School of Agricultural and Life Sciences The University of Tokyo 1‐1‐1 Yayoi, Bunkyo‐ku Tokyo 113‐8657 Japan; ^5^ Research Institute for Agricultural and Life Sciences Tokyo University of Agriculture 1‐1‐1 Sakuragaoka, Setagaya‐ku Tokyo 156‐8502 Japan; ^6^ Biotechonology Center National Chung Hsing University (NCHU) Taichung 402 Taiwan

**Keywords:** drought stress, abscisic acid (ABA), inter‐tissue signaling, inter‐organ signaling, peptide signals, phenotyping

## Abstract

Plant response to drought stress includes systems for intracellular regulation of gene expression and signaling, as well as inter‐tissue and inter‐organ signaling, which helps entire plants acquire stress resistance. Plants sense water‐deficit conditions both via the stomata of leaves and roots, and transfer water‐deficit signals from roots to shoots via inter‐organ signaling. Abscisic acid is an important phytohormone involved in the drought stress response and adaptation, and is synthesized mainly in vascular tissues and guard cells of leaves. In leaves, stress‐induced abscisic acid is distributed to various tissues by transporters, which activates stomatal closure and expression of stress‐related genes to acquire drought stress resistance. Moreover, the stepwise stress response at the whole‐plant level is important for proper understanding of the physiological response to drought conditions. Drought stress is sensed by multiple types of sensors as molecular patterns of abiotic stress signals, which are transmitted via separate parallel signaling networks to induce downstream responses, including stomatal closure and synthesis of stress‐related proteins and metabolites. Peptide molecules play important roles in the inter‐organ signaling of dehydration from roots to shoots, as well as signaling of osmotic changes and reactive oxygen species/Ca^2+^. In this review, we have summarized recent advances in research on complex plant drought stress responses, focusing on inter‐tissue signaling in leaves and inter‐organ signaling from roots to shoots. We have discussed the mechanisms via which drought stress adaptations and resistance are acquired at the whole‐plant level, and have proposed the importance of quantitative phenotyping for measuring plant growth under drought conditions.

## Significance Statement

This review focuses on inter‐tissue signaling, inter‐organ signaling, and stress sensing in complex drought stress responses and tolerance, in which molecular patterns of stress signals are sensed by different sensors and transmitted to other tissues and organs to induce stress responses in total. Recent progress in quantitative phenotyping under drought conditions is also discussed to understand the whole system of complex drought responses and tolerance.

## INTRODUCTION

**Figure 1:**
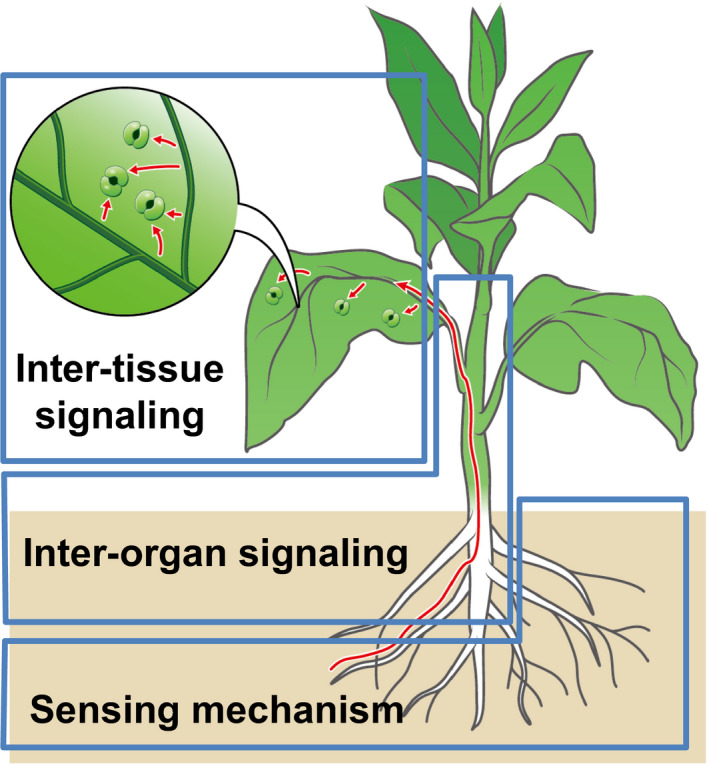
Hierarchy of drought stress responses in plants. Under drought stress conditions, plants perceive water‐deficit via “sensing mechanisms” in roots. Several types of water‐deficit signals are transmitted from roots to leaves via “inter‐organ signaling.” Then, they are distributed between distal tissues in each organ via “inter‐tissue signaling” to adapt to drought stress; for example, abscisic acid functions as an inter‐tissue signal to close stomata and change gene expression in leaves.

Environmental conditions change frequently, both rapidly and incrementally, which affect plant growth and productivity. For example, global warming and climate change can cause droughts, water deficit during which negatively affects plant growth and productivity. Therefore, plants must recognize and respond to environmental changes and adapt to water deficits to survive and grow. Plants have evolved sophisticated systems for adaptation to drought stress to maintain optimal growth under water‐deficit conditions (Gupta et al., 2020). Furthermore, they have developed unique and complex mechanisms connecting various organs and tissues to resist severe environmental stresses. The entire plant body is composed of organs, such as roots, leaves, and stems, and each organ consists of tissues such as epidermal or vascular tissue. The analysis of inter‐tissue and inter‐organ communication systems will provide insights regarding plant responses to water‐deficit conditions, in addition to the cellular mechanism underlying plant responses to dehydration stress (Figure 1).

Abscisic acid (ABA), a phytohormone, functions as an inter‐tissue signal in leaves, one of the above‐ground organs. ABA mediates drought stress responses and resistance by regulating stomatal closure and stress‐responsive gene expression. ABA accumulates mainly in the vasculature of leaves, as the enzymes involved in ABA biosynthesis are expressed in vascular tissues (Chen et al., 2020; Kuromori et al., 2018). In addition, several cellular membrane‐localized ABA transporters are predominantly expressed in vascular tissues. Drought‐induced ABA may be transported from the vasculature to tissues to mediate stomatal movements and gene expression in response to drought stress (Hsu et al., 2021; Kuromori et al., 2018; Munemasa et al., 2015). How tissue‐specific synthesis of ABA and ABA transporter networks control the level of ABA and stomatal closure under drought stress conditions have been discussed subsequently.

Water‐deficit signals are transmitted via inter‐organ signaling from roots to leaves to adapt to drought stress. The vascular system of plants connects the roots and shoots, and plays an important role in integrating stress information from underground organs to aerial organs. Hydraulic signals, Ca^2+^ waves, electric currents, and reactive oxygen species (ROS) mediate drought stress inter‐organ responses, as well as cellular responses (Hsu et al., 2021; Kollist et al., 2019; Takahashi et al., 2020; Zhu, 2016). Hormone‐like peptides act as signaling molecules that mediate inter‐organ stress responses (Gupta et al., 2020; Kim et al., 2021; Li et al., 2021; Takahashi et al., 2018b, 2019, 2020; Thomas and Frank, 2019). These findings suggest that peptides transported by the vasculature integrate water‐deficit stress signals for whole plant‐level communication. Here, we have reviewed the mechanism via which inter‐organ signaling mediates drought stress responses and resistance in plants and the signaling molecules involved.

Under drought stress conditions, plants sense changes in the water‐deficit status of their roots. Drought stress sensing systems are complex. They are stimulated by various stress signals, such as osmotic, ROS, and mechanical stresses, and involve numerous sensing molecules (Takahashi et al., 2020; Yoshida et al., 2021; Zhu, 2016). Environmental stresses are sensed by multiple sensing systems as molecular patterns of stress stimuli, which are transmitted to various tissues to induce specific and sequential stress responses for proper adaptation of plants to complex environmental stresses. We will subsequently discuss the importance of sensing factors of stress signals for correct responses not only at the cellular level, but also at the whole‐plant level. The sensors and signaling patterns mediate the physiological responses of plants to drought stress after sensing the water‐deficit status and downstream stress responses.

The analysis of plant growth under water‐deficit conditions is important for understanding plant responses to drought stress at the whole‐plant level. Toward this, imaging and information technologies have been developed based on artificial intelligence (Dhondt et al., 2013; Mochida et al., 2020; Singh et al., 2016, 2021). Furthermore, quantitative phenotyping enables the analysis of physiological plant responses and assessment of plant water‐use efficiency and drought resistance not only under controlled growth conditions, but also in the greenhouse and field (Singh et al., 2021). In this review, we have described recent advances in plant and crop phenotyping. These new technologies are expected to enhance our understanding of plant responses to drought stress.

## SYNTHESIS AND TRANSPORT OF ABA AND ROLES OF OTHER HORMONES IN INTER‐TISSUE STRESS SIGNALING

Under drought conditions, ABA induces responses that help plants cope with water deficit. As an early drought stress factor, ABA induces guard cells to close the stomata and prevent water shortage. The enzymes involved in ABA biosynthesis are expressed in vascular tissues distant from the guard cells. Therefore, ABA must be transported from vascular tissues to guard cells. Biosynthesis, degradation, and modification of ABA have been investigated (Chen et al., 2020), and various types of ABA transporters have been reported (Takahashi et al., 2020). In response to dehydration, ABA is also rapidly perceived by the guard cells, where it triggers stomatal closure. However, the mechanisms underlying ABA synthesis, metabolism, and transport in drought stress responses are unclear. In this section, we have discussed the ABA synthesis sites, ABA‐mediated inter‐tissue signaling, and function of other hormones in leaves.

### Sites of ABA synthesis in stress responses

**Figure 2:**
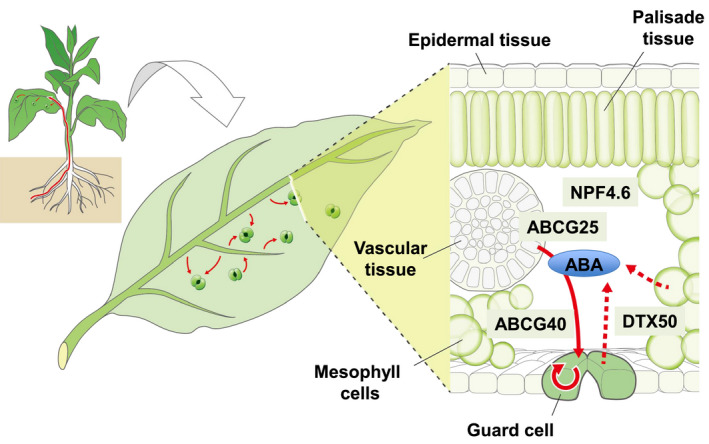
Abscisic acid (ABA) functions as an inter‐tissue signal in leaves. Leaf cross‐section showing vascular tissues (sites of ABA biosynthesis) and guard cells (ABA action sites). In Arabidopsis, the ABA transporters ABCG25, ABCG40, NRF4.6, and DTX50, are expressed in vascular cells and/or guard cells. In the leaf section, the movement of ABA is indicated by solid and dashed arrows.

Control of the water levels in plant bodies is an important environmental response. Being sessile, higher plants sense water deficiency via roots, which are underground organs. This information is transmitted to shoots, including the leaves, which are above‐ground organs, via root‐to‐shoot inter‐organ signals (Christmann et al., 2013; Schachtman and Goodger, 2008) (Figure 2).

ABA is believed to be a major root‐to‐shoot inter‐organ signal. After plants sense water deficiency, ABA is synthesized in the roots and transported to the shoots via xylem flow (Wilkinson and Davies, 2002). However, shoots can also be a source of ABA under stress (Christmann et al., 2007). Foliage‐derived ABA affects drought stress considerably, with leaves being the predominant location of ABA biosynthesis (McAdam et al., 2016).

ABA is synthesized from precursors in plastids via sequential enzymatic reactions. The final three enzymes in the ABA biosynthesis pathway are encoded by *NCED3, ABA2*, and *AAO3* (Finkelstein, 2013). *NCED3* is strongly induced by drought stress, mainly in the vasculature of leaves (Endo et al., 2008), whereas *ABA2* and *AAO3* are expressed under both non‐stress and stress conditions (Cheng et al., 2002; Koiwai et al., 2004; Kuromori et al., 2014a). Although their expression patterns vary, these three genes are predominantly expressed in vascular tissues, which are the major sites of ABA biosynthesis. Drought‐inducible *NCED3* is regulated by the NGA1 transcription factor, which contains a B3 domain (Sato et al., 2018). *NCED3* induction contributes to ABA accumulation in leaves, inducing not only stomatal closure, but also the expression of stress genes.

Guard cells are also sites of ABA biosynthesis and may autonomously support the ABA responses required for stomatal closure (Bauer et al., 2013). They are sensitive to changes in aerial humidity because of their location in the leaf epidermis. In addition, mesophyll cells were found to be the sites of ABA biosynthesis in water‐stressed leaves (McAdam and Brodribb, 2018). In addition to vascular tissues, guard cells and other cells also produce ABA. However, the mechanism via which ABA biosynthesis is coordinated across various tissues in the leaves remains unclear.

In addition to *de novo* biosynthesis, ABA is produced via the hydrolysis of ABA glucose ester (ABA‐GE) by β‐glucosidase. ABA‐GE is a reversibly inactive form of ABA and is believed to act as a storage form of ABA (Lee et al., 2006). ABA‐GE hydrolysis occurs locally in the epidermis of leaf petioles during the early stages of drought stress (Han et al., 2020). Although the physiological effect of ABA‐GE on drought stress responses is unclear, it may contribute to ABA production in leaves (Hussain et al., 2020).

ABA is rapidly catabolized by CYP707As once stress is released. A member of this family, CYP707A3, is predominantly expressed in vascular tissues and regulates ABA accumulation in leaves (Umezawa et al., 2006). Another member, CYP707A1, is preferentially expressed in guard cells and contributes to leaf ABA content, with mutant analyses showing that it regulates the stomatal aperture similar to CYP707A3 (Okamoto et al., 2009). These results also indicated that site‐specific ABA metabolism might be related to ABA responses in leaves.

### Inter‐tissue ABA transport under stress conditions

According to a theory regarding the importance of leaf‐derived ABA over root‐derived ABA, after sensing water depletion in roots, root‐derived signals other than ABA are transmitted to the shoots, including leaves (Kuromori et al., 2014b). Root‐to‐shoot signals induce ABA biosynthesis in leaves by activating *NCED3* expression in vascular tissues, which is the rate‐determining step in ABA biosynthesis (Endo et al., 2008). This suggests that root‐to‐shoot signaling information is transferred via the vascular tissues.

ABA acts as a major inter‐tissue signaling factor in leaves under water stress. In leaves, ABA is transferred to guard cells to close the stomata for preventing water loss. This is followed by changes in gene expression patterns in other cells to cope with water‐deficit stress in tissues (Takahashi et al., 2018a; Yoshida et al., 2021). In general, the sites of action of hormones are distant from their sites of biosynthesis. To act as an inter‐tissue signaling molecule in leaves, ABA is transported to the target guard cells. Indeed, ABA membrane transporters have also been identified (Figure 2).

In Arabidopsis, four membrane proteins, AtABCG25, AtABCG40, AtNPF4.6, and AtDTX50, function as ABA transporters related to ABA inter‐tissue signaling in leaves (reviewed by Kuromori et al., 2018; Shimizu et al., 2021) and are localized to the plasma membrane, indicating that ABA can be exported out of the cells where it is synthesized and imported into cells where it is sensed via the ABA receptor. In addition, mutants of each gene exhibited ABA‐related phenotypes. ABA transporters are regulated at the post‐translational level; for example, the plasma membrane localization of AtABCG25 is regulated by abiotic stress, ABA (Park et al., 2016), and phosphorylated AtNPF4.6, which relocates from the plasma membrane to the membrane of the endoplasmic reticulum (Zhang et al., 2021). In addition, AtABCG22 and AtABCG21 may function in stomatal regulation, although whether they are directly associated with ABA transport across membranes is unclear (Kuromori et al., 2011, 2017).

These transporter genes are mainly expressed in vascular tissues and/or guard cells, corresponding to the sites of ABA synthesis and action, respectively. Furthermore, ABA membrane transporters are categorized as ABA exporters, which mediate ABA export, and ABA importers, which mediate ABA import. ABA is imported into cells for cytosolic proteins that function as ABA receptors responsible for stomatal closure (Ma et al., 2009; Park et al., 2009). Therefore, ABA transport is regulated by membrane transporters in leaf inter‐tissue networks (Kuromori et al., 2018).

Other ABA transporters also control seed germination (Kang et al., 2015). ABA membrane transporters have been identified in non‐model plant species, such as legumes, wheat, and rice (Takahashi et al., 2020). Many types of membrane transporters are involved in ABA transport.

### Roles of other hormones in drought stress responses and tolerance

Hormones are inter‐tissue signals that regulate plant growth under various environmental conditions. ABA, as well as various other phytohormones, are implicated in drought stress tolerance. For example, similar to ABA, methyl jasmonate induces stomatal closure by elevating pH, and the levels of ROS, nitric oxide, and Ca^2+^, leading to the activation of anion channels (Bharath et al., 2021). Jasmonate is involved in the crosstalk between abiotic and biotic stress responses (Huang et al., 2017). Brassinosteroid and auxin responses are related to leaf and root growth under drought conditions (summarized by Gupta et al., 2020). Downstream components of brassinosteroid signaling act by activating ABA signaling. Independent of ABA, brassinosteroid receptors modulate hydrotropic responses in roots and coordinate plant growth and survival under drought stress by promoting the accumulation of osmoprotectant metabolites. In addition, non‐canonical auxin responses modulate root architecture patterning and depth to boost water absorption from the soil, thereby improving drought tolerance (Gupta et al., 2020). In Arabidopsis, auxin‐sensitive Aux/IAA proteins mediate drought tolerance by upregulating glucosinolate levels. The AUX/IAA repressors IAA5, IAA6, and IAA19 are involved in the maintenance of glucosinolate levels when plants are dehydrated. Glucosinolate may function in drought stress responses via ROS, suggesting links between auxin signaling, glucosinolate levels, and drought tolerance (Salehin et al., 2019).

Cytokinin is also involved in drought acclimation/adaptation and in stabilizing plant yield under drought conditions (Hai et al., 2020; Li et al., 2016; Nishiyama et al., 2011). Strigolactone and similar signaling molecules, such as karrikin, function in stomatal responses under conditions of dehydration. For example, shoot‐produced strigolactone induces SLAC1‐dependent stomatal closure by triggering the production of H_2_O_2_ and nitric oxide in guard cells. Moreover, these signaling molecules indirectly affect stomatal closure by positively regulating ABA sensitivity in guard cells (Cardinale et al., 2018). Karrikin and strigolactones function in hormone crosstalk related to drought stress resistance (Li et al., 2017; Mostofa et al., 2018).

The existence of multiple phytohormone functions is indicative of complex plant responses to drought stress in different plant tissues. The terminal phenotypes of drought resistance are achieved via various hormone‐regulatory systems. This reflects the involvement of brassinosteroids, cytokinins, auxin, strigolactones, and karrikin in drought stress resistance, in addition to ABA. Recent reviews have addressed this issue (Hai et al., 2020; Sirko et al., 2021).

## INTER‐ORGAN SIGNALING IN DROUGHT RESPONSES

**Figure 3:**
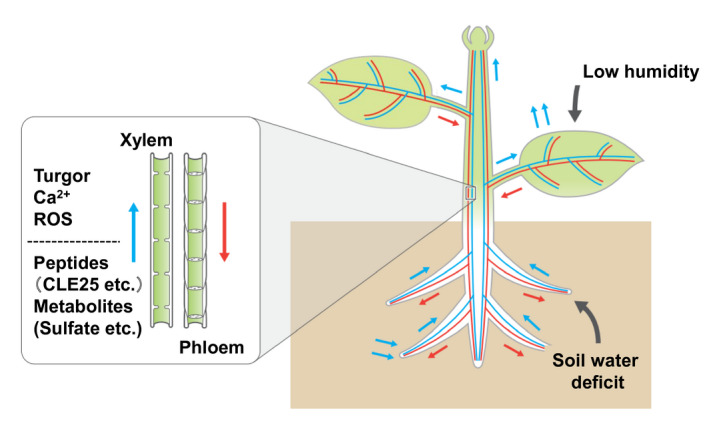
Inter‐organ signaling via the vasculature in drought stress responses. Various inter‐organ signals are transmitted via the vasculature as part of drought stress response. Xylem and phloem are important for inter‐organ signaling. Soil water deficit and low humidity may induce drought stress responses in roots and guard cells, respectively. Osmotic changes, reactive oxygen species (ROS), and Ca^2+^ transients function in stress signaling from roots to leaves. Peptides and metabolites are synthesized in roots in response to drought stress and transported via the xylem to leaves. Among them, the roles of CLE25 peptide have been precisely analyzed (Takahashi et al., 2018b).

Higher plants have developed inter‐organ communication systems that involve various signaling molecules induced by environmental changes. These inter‐organ signals are transmitted via the vasculature to integrate stress responses at the whole‐plant level. Xylem and phloem are important vascular tissues that participate in inter‐organ signaling (Li et al., 2021) (Figure 3). Under drought stress, reduction in water potential in roots informs the plant of soil water deficit (Gupta et al., 2020; Li et al., 2021; Takahashi and Shinozaki, 2019). The water deficit signal is transmitted from the roots to the leaves via the vasculature. Ca^2+^ fluxes, turgor loss, and ROS production are involved in the perception and signaling of dehydration stress responses in the vasculature and guard cells (Chen et al., 2020; Kollist et al., 2019; Soma et al., 2021; Yoshida et al., 2021; Zhu, 2016). After long‐term dehydration, plants accumulate ABA to maintain stomatal closure and induce stress proteins and metabolites to protect organs from dehydration (Thomas and Frank, 2019). Inter‐organ signaling molecules, including peptides and metabolites, activate ABA production at different stages of drought stress response (Li et al., 2021; Takahashi and Shinozaki, 2019; Yoshida et al., 2021). Stepwise responses, including inter‐organ communication, are important for evaluating spatiotemporal drought responses at the whole‐plant level.

### Signaling in early drought responses involving Ca^2+^ and ROS

**Figure 4:**
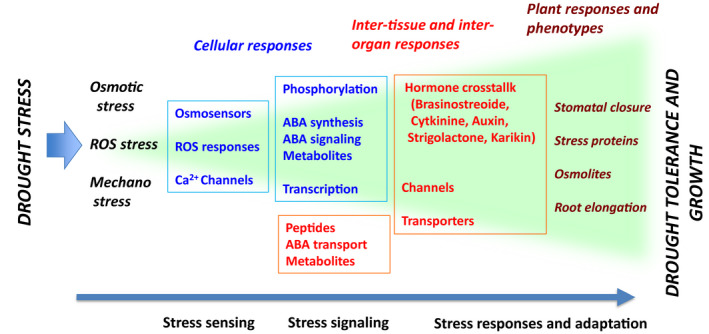
Drought stress signaling network from the perception of stress signals to cellular, inter‐organ, and whole‐plant responses and the acquisition of tolerance. Drought imposes water‐deficit stress on plants at the cellular, organ, and whole‐plant levels. Water deficit induces osmotic stress, oxidative stress, and mechanical stress, which are sensed by osmosensors, reactive oxygen species (ROS) sensors, and Ca^2+^ channels. These intra‐ and inter‐tissue stress signals are mediated by phosphorylation, abscisic acid (ABA), and metabolites. Inter‐organ signaling molecules (peptides such as CLE2 and metabolites such as sulfate) are transported between tissues and organs. Stress signals regulate channels, transporters, transcription factors, and hormones to induce stomatal closure, stress‐responsive gene expression, and osmolyte/stress‐protein synthesis to prevent severe dehydration. After long‐term water‐deficit stress, phenotypic changes in plants such as drought tolerance and growth delay can be monitored precisely using quantitative phenotyping.

Land plants sense water deficiency mainly in the roots by monitoring the water potential in root vasculature. The hydraulic stress signal from root cells is transmitted to the leaves to mediate stomatal closure and promote stress‐inducible gene expression to protect plants from dehydration (Christmann et al., 2013). Ca^2+^ flux across the plasma membrane is activated by various stress signals, including osmotic stress, and Ca^2+^ waves rapidly transmit stress information to distant tissues (Konrad et al., 2018). OSCA1 (REDUCED HYPEROSMOLALITY INDUCED Ca^2+^ INCREASE 1), which encodes a plasma membrane protein, functions as a Ca^2+^ influx channel during osmotic stress response (Choi et al., 2017; Murthy et al., 2018; Yuan et al., 2014) (Figures 3 and 4). *osca1* mutant plants do not regulate stomatal closure and lose more water than wild‐type plants. However, stomatal closure is induced by ABA in *osca1* mutant plants, suggesting a role for OSCA1 in stomatal closure induced by osmotic stress‐mediated Ca^2+^ influx into the stomata. OSCA1.2/CALCIUM‐PERMEABLE STRESS‐GATED CATION CHANNEL 1 (CSC1) functions as a hyperosmolality‐gated Ca^2+^‐permeable channel protein that mediates Ca^2+^ influx (Hou et al., 2014; Liu et al., 2018). Fifteen OSCA family genes have been identified across four clades in Arabidopsis (Yuan et al., 2014). Among them, OSCA1.3 and OSCA1.7 are involved in stomatal immunity downstream of the plasma membrane‐associated cytosolic kinase, BIK1, which is implicated in PAMP‐induced Ca^2+^ influx and stomatal closure (Thor et al., 2020). Therefore, OSCA family membrane proteins mediate Ca^2+^ influx in stress responses and are regulated downstream of receptor‐like kinases. Stretch‐activated Ca^2+^ channels are other candidate drought stress‐sensing factors. In plants, Ca^2+^‐permeable mechanosensitive channels (MCA) 1 and ‐2 sense osmotic changes in the plasma membrane to mediate Ca^2+^ influx and activate downstream cellular signaling (Nakagawa et al., 2007; Nishii et al., 2021). MCA1 is a homolog of yeast MID1, a Ca^2+^‐permeable stretch‐activated channel component. MCA1 and ‐2 are candidate osmosensors that respond to drought stress (Yoshimura et al., 2021).

Oxidative stress signaling is also involved in the systemic response to various environmental stresses. In response to high light and/or heat stress, ROS function not only as local signals in cellular stress response, but also as systemic signals to induce stress responses in remote tissues. Under extreme drought conditions, heat, high light, and water deficit stresses can be severe, suggesting that oxidative stress signaling is induced by drought conditions (Figures 2 and 4). ROS waves are induced by respiratory burst oxidase D (RBOHD) and regulate stomatal closure in stressed leaves. Systemic ROS signaling coordinates responses in leaves to extreme abiotic stresses (Kollist et al., 2019; Zandalinas et al., 2020). HYDROGEN‐PEROXIDE‐INDUCED Ca^2+^ INCREASE (HPCA) is an H_2_O_2_ sensor in guard cells (Wu et al., 2020). HPCA1 is a leucine‐rich repeat receptor‐like kinase (LRR‐RLK), the extracellular domain of which is activated by H_2_O_2_ to induce Ca^2+^ influx into guard cells during stomatal closure. HPCA1 is identical to CANNOT RESPOND TO DMBQ 1 (CARD1), a receptor for quinone perception during haustorium formation in parasitic plants (Laohavisit et al., 2020). HPCA1/CARD1 of the LRR‐RLK subclass VIII‐1 may perform multiple functions in stress responses.

In early drought stress responses, several osmosensing systems recognize dehydration. Moreover, sensing systems in different organs, such as the roots, stomata, and vasculature, may recognize plant dehydration. Early drought stress signals can be recognized by multiple sensing systems based on Ca^2+^ influx and ROS production to activate stress signaling networks, including phosphorylation and downstream gene expression. Molecular patterns of drought stress conditions are important for the recognition of drought conditions to promote plant survival under changing environmental conditions (Figures 3 and 4).

### Roles of peptides in inter‐organ and local dehydration stress signaling

Small peptides function as signaling molecules in plant stress responses. Many novel genes encoding small peptides and non‐coding RNAs have been identified (Takahashi et al., 2019). Proteomic analyses of *Arabidopsis* have revealed novel small peptides predicted to function as regulatory factors in plant development and environmental responses, which are classified as members of the families CLAVATA/EMBRYO‐ SURROUNDING REGION (CLE) and C‐TERMNAL ECODED PEPTIDES (CEP), as well as peptides containing sulfated tyrosine (PSY) (Oh et al., 2018; Ohkubo et al., 2017; Okamoto et al., 2015). CLE peptides are conserved in the plant kingdom (Boschiero et al., 2019; Fletcher, 2020; Goad et al., 2017; Takahashi and Shinozaki, 2019; Whitewoods, 2020). CLE peptides are synthesized as long propeptides, which are then processed into mature peptides of 12–14 amino acids. CLAVATA3 (CLV3) is a key regulator of shoot apical meristem development. In Arabidopsis, the CLE peptide family consists of 27 members that perform diverse functions in development and environmental responses. Among them, CLE25 mediates inter‐organ signaling from roots to shoots in drought stress responses. *CLE25* is mainly expressed in the root vasculature and is upregulated in response to dehydration in root tissues (Figure 3). The *cle25* mutant exhibits a drought‐sensitive phenotype and open stomata. In response to CLE25, *NCED3* expression is induced in the roots and ABA accumulates in the leaves (Takahashi et al., 2018b). Grafting experiments revealed that CLE25 is transported from the roots to the shoots via the vasculature. Two LRR‐RLKs, BARELY ANY MERISTEM (BAM) 1 and 3, recognize CLE25 in leaves and induce *NCED3* expression to close the stomata. Root‐derived CLE25 is an inter‐organ signaling factor that is transmitted via the xylem from roots to shoots and maintains the drought stress response in leaves (Figure 3) by maintaining high ABA levels in leaves. The processes upstream of *CLE25* expression in the root vasculature and downstream of BAM1/BAM3 kinase activity have to be elucidated to understand long‐term responses to dehydration in roots. The NGA1 transcription factor, which has a B3 domain, regulates the dehydration‐induced expression of *NCED3* (Sato et al., 2018). NGA1‐mediated induction of *NCED3* expression may be activated by CLE25. These sequential processes are important for adaptation to long‐term dehydration and maintenance of stress resistance (Figure 3). Vasculature is important for inter‐organ signaling in the regulation of whole‐plant responses (Li et al., 2021). Xylem and phloem transmit mobile signals to distant organs, such as roots and leaves (Figure 3), and mediate upstream and downstream transport of signaling molecules, respectively. In xylem tissue, CLE25 is transported via a transport pattern that differs from that of CLE26 (Endo et al., 2019), indicating the existence of a CLE25‐specific xylem transporter in Arabidopsis. It is necessary to identify the mechanism via which CLE25 and other peptides are transported from the roots to the leaves in response to dehydration stress. Furthermore, the role of the vasculature in coordinating the response to water‐deficit stress status at the whole‐plant level should be investigated.

Other peptides have been shown to be involved in plant drought responses. CLE9 and CLE10 mediate dehydration stress responses in guard cells to regulate stomatal closure. Mitogen‐activated protein (MAP) kinases and SnRK2s are responsible for CLE9‐mediated stomatal closure (Zhang et al., 2019). In addition, CLE9 and CLE10 promote the proliferation of precursors of guard cells and xylem (Qian et al., 2018). Therefore, the tissue‐specific expression of CLE9/CLE10 is critical for their function in environmental responses and development. The genes encoding phytosulfokine precursor (proPSK) and subtilisin‐like protease (SBT) are upregulated in response to osmotic stress (Stuhrwohldt et al., 2021). Overexpression of proPSK and SBTs improved osmotic stress tolerance. SBT3.8 is involved in the posttranslational processing of proPSKs to bioactive PSKs, which also improved drought stress tolerance. Drought‐induced flower drop in tomatoes is regulated by PSK (Reichardt et al., 2020). Mature PSK is formed in response to drought stress by phytaspase 2, an SBT, which then acts in the abscission zone to induce cell wall hydrolases involved in abscission. In rice, OsDSSR1, which encodes a small peptide, has been shown to function in drought tolerance (Cui et al., 2018). Overexpression of OsDSSR1 enhances drought tolerance by inducing the accumulation of compatible osmolytes, superoxide dismutase, and ascorbate peroxidase activities. These recent reports suggest the involvement of different types of small peptides in drought stress response and tolerance.

The *Arabidopsis* genome contains >7000 small open reading frames (sORFs) and sequences for non‐coding RNAs. Transcriptome analyses have shown that the predicted sORFs are expressed under various environmental stresses, such as drought, heat, salinity, and cold stress, as summarized in the HanaDB database (Hanada et al., 2010, 2013; Takahashi et al., 2019). These stress‐responsive sORFs were analyzed by overexpressing them in transgenic *Arabidopsis*. One sORF, named Arabidopsis plant elicitor peptide3 (AtPep3), mediates salt‐stress resistance (Nakaminami et al., 2018). Further analyses of the sORFs regulated by environmental stress will provide insights regarding the functions of various peptides in Arabidopsis. Moreover, some non‐coding RNA genes have been reported to encode peptides (Lauressergues et al., 2015; Ren et al., 2021). Therefore, the functions of these predicted peptide‐encoding genes have to be analyzed. In maize, sORFs and small peptides (sPeptides) were systematically surveyed based on genomic and mass spectrometry data (Liang et al., 2021). Based on these systematic analyses, 9338 sORFs 3–300 nucleotides in length and 2695 sPeptides were identified in the maize genome. PlantPepDB is a manually curated plant peptide database (Das et al., 2020) consisting of 3848 peptides, of which 2821 are experimentally validated at the protein level, 458 at the transcript level, 530 at the predicted level, and 39 based on homology. PlantPepDB is a useful database for comprehensive information on plant peptides.

### Signaling molecules involved in inter‐organ signaling in drought stress responses

Various inter‐organ signaling molecules are important for growth, development, and biotic and abiotic interactions (Li et al., 2021; Thomas and Frank, 2019). Grafting experiments have identified inter‐organ signaling molecules, including proteins, peptides, RNAs, and metabolites (Kurotani and Notaguchi, 2021; Thomas and Frank, 2019). Complex environmental stress signals are transmitted by multiple peptides, proteins, miRNAs, and mRNAs (Figure 3).

Soil drying activates the root‐to‐shoot transport of sulfate. Sulfate, a component of cysteine, induces ABA biosynthesis by activating *NCED3* (Batool et al., 2018). The stomata of the *nced3* mutant did not close after the application of sulfate or cysteine. Sulfate activates NADPH oxidases to induce ROS, which triggers stomatal closure. These findings suggested that sulfate uptake in roots is an inter‐organ signal that activates ABA biosynthesis and stomatal closure in leaves. Cysteine derived from sulfate is involved in ABA synthesis and stomatal closure. The role of sulfate and cysteine in response to soil drying warrants further investigation.

## SENSING OF WATER‐DEFICIT STRESS IN ORGANS

Water‐deficit stress signals are sensed in the sensitive tissues of leaves and roots. In leaves, the dehydration status is sensed by stomata to control plant water status. Stomatal closure decreases water loss from leaves under drought stress conditions and significantly represses CO_2_ uptake to inhibit photosynthesis. In contrast, open stomata control leaf temperature by modulating evaporation. In roots, dry soil causes water deficiency and activates plant drought stress responses. Water deficiency is sensed by root cells, which then initiate drought stress responses (Christmann et al., 2013; Li et al., 2021). In this section, we will discuss the sensing of water‐deficit stress in the stomata and roots.

### Signaling crosstalk in stomatal regulation

Stomata play important roles in CO_2_ and O_2_ exchange during photosynthesis. Moreover, stomata manage the water status of plants. Stomatal responses are regulated by various environmental stimuli, such as water status, light intensity and wavelength, humidity, CO_2_ level, and pathogen infection (Hsu et al., 2021; Yoshida et al., 2021). Furthermore, environmental stresses such as ABA, ROS, and Ca^2+^ were found to regulate stomatal responses. ABA induces rapid stomatal closure via the canonical ABA receptor signaling machinery in response to early stages of drought stress. This machinery includes the PYR/PYL/RCSR ABA receptor, protein phosphatase 2C (ABI homolog), and SnRK2 protein kinases, which activate downstream transporters to regulate stomatal responses. The ABA receptor‐signaling machinery has been reviewed elsewhere (Chen et al., 2020; Cutler et al., 2010). Ca^2+^/ROS signals also regulate stomatal closure by modulating the activity of channel proteins, including SLAC1 and KAT1, as reviewed by others (Hsu et al., 2021; Yoshida et al., 2021).

### Sensing of water‐deficit status in the roots and vasculature

The water‐deficit status of soil is sensed in roots, of which vascular tissues respond to hydraulic stress and low water potential. Hydraulic stress signaling in roots is believed to be an early drought stress response (Figures 3 and 4). However, the mechanism via which hydraulic stress is sensed in the roots is unclear. Histidine kinases function as osmosensors in yeast and bacteria, including cyanobacteria. In yeast, Sln1p, an osmosensor, acts upstream of the HOG1 MAP kinase pathway. In Arabidopsis, histidine kinase 1 (ATHK1/AHK1) is a functional homolog of yeast Sln1p, which also functions as an osmosensor (Urao et al., 1999). Analyses of mutant and overexpression strains have revealed that ATHK1/AHK1 positively regulates drought stress tolerance (Tran et al., 2007; Wohlbach et al., 2008). However, ABA responses or stomatal closure was not affected in *ahk1* mutant (Kumar et al., 2013; Sussmilch et al., 2017). These inconsistent data indicated the existence of complex osmosensing systems in plants, which include AHK1 and its downstream signaling pathway, along with MAP kinases. Plant MAP kinases are activated by abiotic and biotic stress signals to control downstream events, including gene expression and physiological responses (Lin et al., 2021). In contrast, AHK2, AHK3, and AHK4 (CRE1) are cytokinin receptors that function as negative regulators of drought response (Nishiyama et al., 2013). AHK4 (CRE1) complements yeast *snl1* mutants in the presence of cytokinin, indicating its osmosensing ability (Reiser et al., 2003).

Root hydrotropic response is important for avoiding dry soil and obtaining water for growth. *MIZU‐KUSSEI1* (*MIZ1*), involved in hydrotropic response, is expressed in the epidermis, cortex, and lateral root cap (Dietrich et al., 2017; Kobayashi et al., 2007; Moriwaki et al., 2012, 2013; Takahashi et al., 2002). *MIZ1* is induced by light in the root cap, and its expression is significantly low in plants harboring the mutant HY5 transcription factor (Lee et al., 2007). HY5 functions as an inter‐organ signal from shoots to roots, mediates light‐responsive root growth (Chen et al., 2016), and stimulates the MIZ1‐mediated hydroponic response to increase water uptake. The role of HY5 in drought stress avoidance requires further analysis. MIZ1 also functions in the regulation of inter‐organ Ca^2+^ signaling in root tip cells (Shkolnik et al., 2018).

Root growth, morphogenesis, and architecture are genetically regulated for the efficient absorption of water and nutrients from soil. The long and deep phenotype of roots is regulated via quantitative traits, and the DEEPER ROOTING1 (DRO1) locus has been analyzed to identify the related genes in rice (Uga et al., 2013). Root branching is associated with water availability and seed production, a phenotype known as hydropatterning. Rice *DRO1* encodes an unknown factor related to auxin signaling. In Arabidopsis, AUXIN RESPONSE FACTOR7 (ARF7) initiates lateral root growth. On the dry side of roots, ARF7 is modified by a small ubiquitin‐like modifier (SUMO) and inactivated by the repressor IAA3, triggering hydropatterning (Orosa‐Puente et al., 2018; Yoshida et al., 2021).

The vasculature transports nutrients and signals from the roots to the shoots. Water deficit is sensed in the vasculature with a reduction in the water potential (Figure 3). Xylem, phloem, phloem companion cells, and epidermal cells recognize the reduction in water potential caused by drought stress (Endo et al., 2019; Li et al., 2021; Lucas et al., 2013). However, it is not known which molecules in these cells of the vascular tissues that sense the reduction in water potential.

The time course of drought stress responses, their molecular networks in complex stress signaling, and drought tolerance phenotypes are schematically described in Figure 4. Spatiotemporal responses to drought stress are complex processes involving molecular patterns of different types of stress signals, including cellular and inter‐organ responses induced by osmotic, ROS, and mechanical stresses, which are integrated to induce drought stress responses and tolerance at the whole‐plant level.

## HIGH‐THROUGHPUT PHENOTYPING OF DROUGHT‐STRESS RESPONSES AND TOLERANCE

In the previous sections, we have discussed inter‐tissue and inter‐organ signaling, and sensing in drought tolerance. To understand how stress resistance is acquired at the whole‐plant level via signal transduction at various spaces and scales, it is necessary to observe the whole‐plant phenotype over time in a system that allows precise and highly reproducible analysis. Therefore, this section introduces high‐throughput phenotyping technologies, which have developed at a rapid pace in recent years, to understand comprehensively how signaling at various levels in drought stress response affects the whole plant. In addition, high‐throughput phenotyping techniques in the laboratory under controlled conditions, as well as those in the field under highly uncertain conditions, will be discussed.

### High‐throughput phenotyping of drought stress responses and tolerance in the laboratory

Environmental factors in the field are complex and unpredictable, necessitating laboratory studies under controlled environmental conditions. High‐throughput phenotyping has progressed rapidly, resulting in automation and non‐destructive analyses (Dhondt et al., 2013). In addition, non‐destructive analysis, such as imaging analysis, can be combined with machine and deep learning to improve accuracy and efficiency (Li et al., 2020; Singh et al., 2016, 2021). The analysis of the resulting big data has been enhanced by various innovations (Tardieu et al., 2017). In addition, multiomics analysis, which integrates data from multiple omics methods, is progressing (Mochida et al., 2020).

In this subsection, we have first focused on high‐throughput phenotyping of *Arabidopsis thaliana*, which is essential for the analysis of systems regulating drought stress responses and tolerance. Automated phenotyping platforms for *A. thaliana*, such as PHENOPSIS (Granier et al., 2006), WIWAM (Skirycz et al., 2011), Phenoscope (Tisne et al., 2013), Phenovator (Flood et al., 2016), and RIPPS (Fujita et al., 2018) have been developed.

The PHENOPSIS platform (INRA, France) has been used to identify loci involved in natural variation and water‐deficit responses in *Arabidopsis* accessions and inbred lines (Bac‐Molenaar et al., 2016; Ghandilyan et al., 2009; Rymaszewski et al., 2017; Schmalenbach et al., 2014; Tisne et al., 2010; Vasseur et al., 2014; Vile et al., 2012), and to evaluate the function of genes such as *RD20* (Aubert et al., 2010) and *SMR1* (Dubois et al., 2018) in drought stress. PHENOPSIS also enables the investigation of plant–microbe and abiotic–biotic stress interactions (Berges et al., 2018, 2020; Bresson et al., 2013).

WIWAM (VIB, Belgium) has been used to study the response of *Arabidopsis* to mild drought stress (Clauw et al., 2015, 2016; Dubois et al., 2017). The comparative analysis of *Arabidopsis* and its drought‐tolerant relatives revealed that important differences in drought responses among *Brassica* plants are likely to occur in downstream signaling and response networks rather than in initial water deficit‐sensing mechanisms (Marín‐de la Rosa et al., 2019).

The Phenovator is a benchtop high‐throughput photosynthesis phenotyping platform that has been used in studies on *Arabidopsis*. Unlike PHENOPSIS and WIWAM, rockwool blocks, instead of soil‐filled pots, are used for hydroponics, facilitating the analysis of regulatory systems responsible for drought stress responses in up to 1440 *Arabidopsis* plants (Flood et al., 2016). Benchtop phenotyping platforms may not be able to reproduce phenotypes due to uncontrolled noise sources, leading to micro‐ and macro‐environmental variability (Massonnet et al., 2010). To overcome these problems, conveyorized phenotyping platforms such as Phenoscope (France) and RIPPS (RIKEN, Japan) have been developed (Fujita et al., 2018; Tisne et al., 2010). Rotation of the pot significantly reduces the small environmental perturbations that exist even under well‐standardized conditions. Phenoscope analysis with *Arabidopsis* accessions showed that mild drought stress did not exert any epigenetic effects across generations (Van Dooren et al., 2020). The Phenoscope can analyze up to 735 plants, unlike 120 plants that can be analyzed using RIPPS. RIPPS, however, uses fewer root system constraints and larger pots, a system that minimizes water evaporation; furthermore, various state‐of‐the‐art equipment for drought analysis is used (Fujita et al., 2018). The RIPPS platform revealed that the ABA transporter, ABCG25, improves water‐use efficiency and drought tolerance (Kuromori et al., 2016) and that *NCED3* (involved in ABA biosynthesis) and *CYP707A* (involved in ABA catabolism) are important for water‐use efficiency (Fujita et al., 2018). Thus, RIPPS enables the investigation of phenotypes of *Arabidopsis* mutants and stress‐exposed plants, and will promote the analysis of drought‐response mechanisms, particularly with the development of imaging systems and water/nutrient delivery systems. It is also expected to facilitate the mathematical analysis of drought‐response mechanisms.

The drought responses and tolerance of crops have been investigated using indoor high‐throughput phenotyping platforms. Scanalyzer 3D is a typical conveyor‐type high‐throughput phenotyping platform developed by LemnaTec GmbH (Aachen, Germany) and has been used to study drought responses and tolerance in barley (Chen et al., 2014; Neumann, 2015), sorghum (Neilson et al., 2015), *Seteria* (Fahlgren et al., 2015), rice (Campbell et al., 2020; Duan et al., 2018), and wheat (Bruning et al., 2019). As part of the Montpellier plant phenotyping platforms (Cabrera‐Bosquet et al., 2016; Tardieu et al., 2017), the PhenoArch platform was used to analyze the growth of maize ears and silique under drought conditions (Brichet et al., 2017), drought response in terms of leaf water potential and transpiration of grape vines (Coupel‐Ledru et al., 2014), and the water‐use efficiency of apple trees (Lopez et al., 2015). A high‐throughput rice phenotyping facility with image analysis pipeline at Huazhong Agricultural University in China (Yang et al., 2014) was used to quantify the dynamic response of rice to drought (Duan et al., 2018) and reveal the genetic architecture of drought resistance in rice (Guo et al., 2018) and cotton (Li and Shen, 2020). In addition, root phenotyping is being studied using X‐ray computed tomography (CT) and magnetic resonance imaging (Atkinson et al., 2019). A non‐destructive X‐ray CT method has been developed to analyze the effects of high temperature and drought stress on potato tubers over time (Van Harsselaar et al., 2021). OpenSimRoot, a functional structural three‐dimensional plant model, enables the mathematical description of root growth and function (Postma et al., 2017). This software can be applied to model three‐dimensional images of roots in soil using magnetic resonance imaging and X‐ray CT. Analysis with OpenSimRoot revealed that the metaxylem morphology interacts with root system depth to regulate water use under drought stress (Strock et al., 2021). A biological organic electrochemical transistor sensor‐based method (Bioristor) was developed to analyze the response of tomato plants to drought (Janni et al., 2019). An Internet of Things‐based pot system (iPOTs), in which the soil water condition can be adjusted via the application of optional treatments, was developed to monitor rice growth under drought stress conditions (Numajiri et al., 2021). The physiological state of the plant can be continuously monitored by embedding the Bioristor device into the tomato stem (Janni et al., 2019). Bioristors can detect drought stress‐induced changes in ion concentrations in the sap, enabling detection of the onset of drought stress immediately after the initiation of defense responses (Janni et al., 2019). In future, further developments in smart plant sensors based on nanobiotechnology (Giraldo et al., 2019) and in technologies for biomolecular detection based on wearable materials integrated with synthetic biology sensors (Nguyen et al., 2021) are expected to advance plant phenotyping considerably.

In pot‐based phenotyping of drought responses, the use of pots may limit root elongation and growth, and the high frequency of deficit irrigation may lead to uneven distribution of water in the soil, affecting plant growth, root distribution, water and nutrient uptake, and root–shoot interactions (Puertolas et al., 2017; Turner et al., 2019). The use of larger pots such as RhizoTubes (Jeudy et al., 2016), rooting columns (Gebre and Earl, 2020), and rhizotrons (Belachew et al., 2019; Canales et al., 2019) has been examined as possible solutions to these problems despite certain limitations. These problems associated with pot‐based phenotyping have not been observed in field phenotyping. However, field work is hampered by larger problems caused by uncontrollable environmental fluctuations. The weaknesses and strengths of laboratory and field phenotyping have to be recognized to identify drought‐response mechanisms and develop drought‐tolerant crop varieties.

### Phenotyping to evaluate drought stress tolerance in the field

In the field, large experimental plots enable the collection of large amounts of data using unmanned aerial vehicles and remote sensing. In addition, data science is required to extract relevant information. Automated, non‐destructive, and image‐based high‐throughput phenotyping now allows acquisition of temporal data beyond the terminal phenotype with lesser effort than manual phenotyping (Li et al., 2020). Drought research using high‐throughput phenotyping is currently being developed, the progress of which is outlined below.

In wheat, Phenocart, a portable field phenotyping system (Crain et al., 2018), was used to assess simultaneously the normalized difference vegetation index (NDVI) and canopy temperature of 1170 lines grown under drought or high‐temperature conditions, and to evaluate several genomic selection models (Crain et al., 2018). Durum wheat accessions were grown and phenotyped under different irrigation conditions (Condorelli et al., 2018; Gomez‐Candon et al., 2021). A cost‐effective proximity‐sensing cart equipped with an infrared thermometer, ultrasonic transducer, multiple spectral reflectance sensors, weather station, and RGB camera was used to evaluate upland cotton (Thompson et al., 2018). A field phenotyping platform consisting of a high‐throughput phenotyping system with a gantry frame equipped with various sensors and integrated into a large‐scale automated rainfall shelter facility was constructed to investigate water and nitrogen stress responses in wheat (Beauchene et al., 2019). This environmental management system is promising, as it enables accurate comparative evaluation in the field. As environmental control other than that of soil moisture is dependent on environmental conditions and is expensive, the use of field systems in combination with laboratory studies will accelerate phenotypic analysis.

## SUMMARY AND FUTURE PERSPECTIVES

Gene expression and signal transduction related to the plant drought stress response have been studied at the cellular and molecular levels in model plants and crops, and the functions of genes involved in stress tolerance have been analyzed in transgenic and mutant plants. In addition, the mechanisms underlying the induction of gene expression by drought stress and its regulation by ABA and protein phosphorylation have been analyzed. Stomatal closure due to water deficiency has been evaluated at the cellular and molecular levels, revealing complex regulatory systems mediated by protein phosphorylation. The molecular mechanisms underlying stomatal responses to dehydration, CO_2_, and light crosstalk, integrating complex responses to environmental changes.

The molecular transmission of stress signals in tissues and organs was analyzed. Research on plant responses to abiotic stresses has shifted from intra‐cellular to inter‐tissue or inter‐organ systemic regulation (Figure 1). To understand drought stress responses and tolerance in the whole plant, inter‐tissue transmission of stress signals has been analyzed with respect to ABA. For example, several types of transporters are involved in ABA transport between the vascular bundle, stomata, and the entire leaf (Figure 2). Furthermore, the induction of stress‐responsive gene expression and acquisition of stress tolerance via the systemic transport of ABA and stress signals have been investigated. Analyses of regulatory genes related to ABA transport have been performed, and research on inter‐tissue communication is underway.

Stress signals due to soil water loss are sensed by the roots and transmitted to the leaves. The roles of turgor pressure, Ca^2+^, and ROS have been analyzed in inter‐organ signal transduction from the roots to the leaves. Inter‐organ signal transduction from roots to leaves is mediated by the transport of peptides and metabolites via the vasculature, particularly the xylem, which involves various molecular mechanisms (Figure 3). The transport of these inter‐organ signaling molecules will be investigated based on functional analyses of the vascular transport systems. Novel peptide‐coding genes have been identified during the analysis of genomes and non‐coding RNAs. Predicted sORF and proteomics (PeptideAtlas/Arabidopsis; http://www.peptideatlas.org/builds/arabidopsis/) databases are publicly available, and additional functional analysis of predicted peptides will reveal novel regulatory systems.

Drought stress or water‐deficit stress causes complex physiological responses in different plant organs. Moreover, complex drought stress signals are sensed to induce complex molecular patterns of intra‐ and inter‐cellular stress signals, which are recognized by different cellular sensors to induce correct responses required for survival under drought stress conditions (Figure 4). Water‐deficit stress signals are also multilaterally sensed in the leaves and roots. In leaves, the dehydration status is sensed by stomata to control the water status. Stomatal closure decreases water loss from leaves under drought stress conditions. In roots, dry soil causes water deficiency, which is sensed mainly by the vascular tissues of roots to activate inter‐organ stress responses from roots to leaves. Water deficiency is sensed by root vascular cells, which initiates drought stress responses in all plant organs. Integration of these stress responses in different organs is necessary for proper responses of the entire plant (Figures 3 and 4).

Non‐destructive and quantitative analyses of plant phenotypes have been enabled by developments in imaging and data analyses. Plant development under stress conditions can be analyzed using automatic phenotyping systems under controlled environmental conditions. Imaging systems using high‐performance cameras allow monitoring of plant growth under various environmental conditions. For example, leaf temperature can be measured using an infrared camera, and plant water status can be measured using a near‐infrared camera to monitor dehydration. By continuously monitoring the water‐deficit status of plants, it is possible to measure quantitatively the response of plants to water loss in terms of plant growth, stomatal closure, and water status. Transcriptomic and metabolomic analyses facilitated the identification of novel gene sets using mutant plants and natural ecotypes. Furthermore, genome‐wide association studies of mutant strains can reveal novel genes involved in water‐use efficiency and dehydration resistance. In addition, the effects of drought stress on plants can be investigated in the reproductive stage, such as flower formation and seed maturation. The drought responses of plants in the field were predicted using the data obtained from quantitative phenotypic analysis. In the future, these data will be related to crop growth and the acquisition of tolerance during drought conditions, along with meteorological data. Further progress in information technology and data science will enable the use of big data in research on the environmental responses of plants. Interdisciplinary phenomics research on plant development, such as vegetative growth, flowering, and seed formation, will be facilitated by further technological developments and the anticipated availability of more plant and crop biological resources.

## AUTHOR CONTRIBUTIONS

TK and KS wrote and edited a large part of this review article. MF mainly wrote the phenotyping section and FT wrote the inter‐organ signaling section. All the authors contributed to the edition and revision of this article.

## CONFLICT OF INTEREST

The authors declare that they have no competing interests.

## Data Availability

This is a review article. All relevant data can be found within the manuscript and its supporting materials.
